# Exploring the content of epilepsy fellowship program websites: an analysis of information available to applicants

**DOI:** 10.1186/s12909-024-05612-x

**Published:** 2024-06-27

**Authors:** Shayan Marsia, Alishba Kamran, Sayed Mustafa Mahmood Shah, Rameez Ali Merchant, Shan E. Abbas

**Affiliations:** 1 Department of Neurology, Spectrum Health, Michigan State University, Michigan, USA; 2 Dow University of Health Sciences, Karachi, Pakistan; 3 Department of Neurology, University of Missouri-Columbia, Missouri, USA; 4 Department of Neurosurgery, University of Michigan, Michigan, USA; 5 Corewell Health West & Corewell Health Grand Rapids, Michigan State University, Michigan, USA; 6 Corewell Health West, Michigan, USA

**Keywords:** Epilepsy, Fellowship program, Applicant resources, Program evaluation, Medical education, Website analysis

## Abstract

**Background:**

Program websites are essential resources in the process of residency and fellowship application. We evaluated the information furnished on these resources by Epilepsy fellowship programs. The extent of information provided was compared across geographic zones, academic affiliation, and national ranking.

**Methods:**

A list of Epilepsy fellowship programs was derived from the Fellowship and Residency Electronic Interactive Database (FREIDA). Links to program websites were obtained directly from FREIDA or using Google’s search engine. Online data was categorized to reflect program information, education, recruitment, compensation, epilepsy center-specific information, and social media presence. Data points under each category were collected to develop a standardized scoring system. The frequency of criterion present was compared across geographic zones, academic affiliation, and national ranking using parametric and non-parametric statistical tests. Significance was determined at a p-value ≤ 0.05 for all cases. The study utilized IBM SPSS version 28 and Python 3.11.3.

**Results:**

We analyzed 80 Epilepsy fellowship programs. The most reported feature was the program director’s name and email (100.0%). The least reported features included board pass rates (1.3%), preparatory boot camp (8.8%), and post-fellowship placements (11.3%). Programs were found to be well-represented on X (88.8%), Facebook (81.3%), and Instagram (71.3%). Most (85.0%) of the programs were searchable through Google. The scores for program information, education, recruitment, compensation, epilepsy center-specific information, and social media visibility did not significantly vary based on location, academic affiliation, or rank status.

**Conclusions:**

Our results demonstrate that despite an online presence, there is much room for improvement in the content available to the applicant. To improve the Match process and attract a roster of well-informed fellows, Epilepsy fellowship programs should furnish program websites with up-to-date information relevant to program information, education, recruitment, compensation, and epilepsy center-specific information.

## Background

Epilepsy is one of the most common disorders of the nervous system [1]. Epileptology, a subspecialty within neurology, specializes in the treatment of epilepsy, particularly complex cases that are resistant to basic treatments. Advanced training in Epilepsy requires a one to two-year fellowship following residency in Neurology or Child Neurology. The curriculum primarily emphasizes clinical management and surgical planning and offers exposure to advanced diagnostic techniques such as EEG and neuroimaging. Many subspecialties like epilepsy have historically not been covered by a formal match system, which pairs medical professionals with their training programs, due to several reasons. Challenges include establishing standardized training protocols for newer or highly specialized fields, limited accredited fellowship programs, and lower demand relative to broader specialties. In 2017, Vidaurre and Campbell advocated a formal matching system for trainees applying to Epilepsy and Clinical Neurophysiology fellowships. They cited several advantages, such as facilitating the process, promoting a more structured environment, and encouraging training programs to improve their infrastructure to attract top applicants [2].

Recently, the National Resident Matching Program (NRMP) has implemented a Match for epilepsy and clinical neurophysiology, two closely related subspecialties of neurology offered by a considerable number of institutions [3]. Within this matching system, eligible candidates must research various programs and decide which programs to apply for and rank based on their preferences after interviewing.

The American Medical Association’s (AMA) Fellowship and Residency Electronic Interactive Database Access (FREIDA) [4] is a reliable resource for candidates seeking information about different programs. However, program websites are often the primary resource for information about fellowship opportunities. These websites provide additional insights into a program’s contact details, application requirements, values, mentorship, and research opportunities. Several studies have shown that program websites influence applicants’ decisions to apply to a specific program: In a study by Gaeta et al., 78% of Emergency Medicine applicants said that a program website’s content influenced their choice to apply to a specific program [5]. Similarly, 56% of Stanford Anesthesia residency program applicants surveyed by Chu et al. reported doing research on the program website before they chose to apply to it [6].

Despite the importance of program websites, several studies have found that they often lack accessibility and comprehensiveness. For example, Khan et al. evaluated the online profiles of 221 US-based cardiology fellowship programs and found that only 25 (11.3%) were fully current [7]. Trehan et al. found that only 64% of 81 Hand Surgery fellowship programs had sufficient online information for residents to complete the application process independently [8]. Hsu et al.‘s study on 84 Neuroradiology fellowship program websites found that basic information like program descriptions and contact details were commonly available. In contrast, details like interview day itinerary, meal allowance, and post-fellowship placement were less frequently provided [9].

Our analysis aims to evaluate the accessibility and comprehensiveness of information available on Epilepsy fellowship program websites. We hypothesize that there is a paucity of detail regarding ACGME (Accreditation Council for Graduate Medical Education)-accredited programs’ education, recruitment, and compensation.

## Methods

In July 2022, we obtained a list of 93 Epilepsy fellowship programs from the Fellowship and Residency Electronic Interactive Database (FREIDA) [4]. We derived program website links available on FREIDA webpages, using the inclusion criteria that the links were accessible, functional, and provided relevant program information. In cases where a website link was not available on FREIDA, we conducted a Google search to find information about the program and considered it for inclusion if found. Programs were excluded if the website link provided on FREIDA was not functional or accessible, and no alternative link could be found through a Google search. Additionally, programs were excluded if their website links failed to provide sufficient dedicated information about the fellowship. A total of 80 programs were included.

To determine the prominence of each program within search results, we conducted Google searches for each program using the search term “ACGME listed title of institution + epilepsy fellowship” (e.g., “The University of Alabama at Birmingham + epilepsy fellowship”). Sponsored links were excluded, and the search findings were documented.

Two independent reviewers (A Kamran and SMM Shah) accessed and examined the program website and FREIDA webpage. A scoring system was created based on the ACGME common program requirements, the ACGME program requirements for graduate medical education in epilepsy, and general fellowship website criteria found in prior literature. We collected general information about the programs, such as program size, mission statement, and diversity information. We also evaluated the categories of recruitment, education, compensation, and social media content. Additionally, we collected epilepsy center-specific information, including National Association of Epilepsy Centers (NAEC) Level designation, Epilepsy Monitoring Unit (EMU) rotation description, the availability of experience in Deep Brain Stimulation (DBS) and Responsive Neurostimulation (RNS), and Intraoperative Monitoring (IOM). Furthermore, we evaluated website features indicating its update, including the availability of the following information: 2020–2022 fellows listed, 2021 copyright, 2021 fellow catalog, 2021–2022 fellows listed, 2022 copyright, 2022 application deadline, and 2022 stipend information. A total of 72 criteria were evaluated (Table 2). The presence of each specific criterion received a score of 1 point and the absence or insufficiency received a score of 0. In cases of disagreements between the two independent reviewers, a final verdict was reached by consensus with a third reviewer (S Marsia). Inter-rater agreement for each checklist item was evaluated by the chance-corrected measure of agreement, Cohen’s κ. The Kappa value obtained was 0.87.

We categorized the programs based on geography (U.S. Census Bureau designated divisions), program type (community- or university-based), and U.S. News ranking. The programs were categorized into four geographic regions: Northeast (*n* = 26 [Maryland, Pennsylvania, Delaware, New Jersey, New York, Connecticut, Massachusetts, Vermont, Rhode Island, New Hampshire, Maine, and the District of Columbia]), Midwest (*n* = 21 [Nebraska, Kansas, Minnesota, Iowa, Missouri, Wisconsin, Illinois, Michigan, Indiana, Ohio, South Dakota, North Dakota]), West (*n* = 24 [New Mexico, Wyoming, Idaho, Montana, Colorado, Washington, Oregon, Nevada, Utah, Arizona, Alaska, California, Hawaii]), and South (*n* = 22 [Virginia, Kentucky, Arkansas, Oklahoma, Texas, North Carolina, South Carolina, Georgia, Florida, Louisiana, Mississippi, Alabama, Tennessee]). We obtained a list of the top 50 hospitals specializing in Neurology and Neurosurgery from US News and World Report, published in July 2022 [10].

We used IBM SPSS version 28 and Python 3.11.3 for data analysis. Figures were created using the Seaborn library in Python. Mean values, standard deviations, median values, and IQR were calculated for each category based on the number of criteria fulfilled. To assess differences between scores for each category and geographical region, program types, and rankings, we conducted Kruskal-Wallis tests, Mann-Whitney U tests, ANOVA, and independent sample t-tests. During analysis, we excluded the “other” category in program types due to a small sample size (< 5) [11], and we compared the means of community- and university-affiliated programs. Significance was determined at a p-value ≤ 0.05 for all cases.

## Results

Data on 80 programs offering fellowship training in Epilepsy was collected. Table 1 presents the characteristics of these programs. They were evenly distributed across four geographic zones: Midwest (23.75%), Northeast (28.75%), South (22.50%), and West (25.00%). The majority of programs (78.75%) were university-based, and 43.75% of them were ranked among the top 50 hospitals for neurology and neurosurgery in the US.

**Table 1:** Program characteristics

Program Characteristics	*n* (%)
**Location**	
Midwest	19 (23.75)
Northeast	23 (28.75)
South	18 (22.50)
West	20 (25.00)
**Academic affiliation**	
University-based	63 (78.75)
Community-based university affiliated	16 (20.00)
Other	1 (1.25)
**US top 50 ranking**	
Ranked	35 (43.75)
Unranked	45 (56.25)
**Available general information**	
Program size	72 (90.00)
Mission statement	79 (98.75)
Diversity information	58 (72.50)

Table 2 depicts the frequency of general information, recruitment, education, compensation, epilepsy center features, social media, and update information.

**Table 2:** List of criteria and frequency of available information

Feature	Percentage
**General information**	
Mission statement	98.8
Program size	90.0
Diversity information	72.5
**Recruitment**	
Program email address	100.0
Program directors’ name	100.0
Program directors’ email	100.0
Program description	98.8
Program directors’ contact number	95.0
Searchable on google	93.8
Program contact number	93.8
Application requirements	92.5
Number of fellowship positions	91.3
1st search on Google (excluding ads)	85.0
Application deadline	55.0
Visa information	53.8
Link to application	51.3
Information about the area	48.8
Interview dates	41.3
Fellow life, excursion trips and extra curriculars	41.3
IMG requirements	36.3
Program directors’ message	15.0
Legal policies	12.5
Board pass rates	1.3
Interview day itinerary	0.0
**Education**	
Clinical sites/Affiliated hospitals	100.0
Fellow research or quality improvement (QI) activity	91.3
Current Faculty listing	85.0
Curriculum	78.8
Didactics	70.0
Academic conferences	65.0
Supervision	57.5
Current Fellow listing	41.3
Rotation schedule	38.8
Journal club	36.3
Case load	33.8
Call schedule	32.5
Professional development fund	25.0
Alumni listing	22.5
Association to professional societies	15.0
Responsibility progression	12.5
Post fellowship placement	11.3
**Epilepsy specific content**	
Epilepsy surgery	80.0
Epilepsy monitoring unit (EMU) rotation/number of beds	72.5
Additional year/research track	70.0
National Association of Epilepsy Center (NAEC) Level	50.0
Responsive neurostimulation (RNS)	46.3
Intra-operative monitoring (IOM)	38.8
Specified number of rotations for pediatric electroencephalogram (EEG)	37.5
Deep brain stimulation (DBS)	26.3
Preparatory bootcamp	8.8
**Compensation**	
Salary	61.3
Benefits	61.3
Vacation days	57.5
Insurance	56.3
Meal Allowance	52.5
Car parking	50.0
Moonlighting	48.8
Debt management	48.8
Housing	45.0
**Social Media**	
X	88.8
Facebook	81.3
Link to website from social media profile	78.8
Instagram	71.3
LinkedIn	57.5
**Update criteria**	
Last updated	100.0
2022 copyright	78.8
2022 stipend information	55.0
2022 application deadline	51.3
2021–2022 fellows listed	20.0
2021 fellow catalog	17.5
2020–2021 fellows listed	13.8
2021 copyright	2.5

The most reported features in recruitment included the program director’s name and email (100%), program description (98.8%), and program director’s contact number (95.0%). On the other hand, board pass rates (1.3%), legal policies (12.5%), and program director’s message (15.0%) were the least reported items. No interview day itineraries were found (0.0%).

For educational information, the most reported items included clinical sites/affiliated hospitals (100.0%), fellow research or quality improvement (QI) activities (91.3%), and current faculty listing (41.3%). The last reported items included post-fellowship placement (11.3%), responsibility progression (12.5%), and association with professional societies (15.0%).

Most programs provided a description of the epilepsy center features available to fellows, including details pertaining to the program’s epilepsy surgical program (80%), EMU rotation description (72.5%), and information on additional year/research tracks (70.0%). However, IOM (38.8%), pediatric electroencephalography (EEG) (37.5%), DBS procedures (26.6%), and preparatory boot camps (8.8%) were less likely to be reported.

The most reported factors related to compensation included salary and benefits (61.3%), vacation days (57.5%), and insurance (56.3%). Housing (45.0%) and moonlighting (48.8%), and debt management (48.8%) were the least likely to be reported.

The top three social media platforms used by the programs included X (formerly Twitter) (88.8%), Facebook (81.3%), and Instagram (71.3%). 78.8% of programs provided a functioning link to the program website on at least one of its social media profiles. The last information update was mentioned on the FREIDA web pages (100.0%). The 2022 application deadline and stipend information were provided by 51.3% and 55.0% of programs, respectively.

**Fig. 1:**
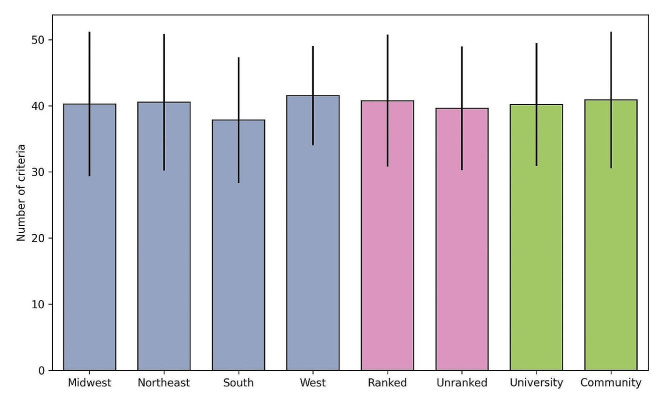
Number of criteria mentioned on programs’ websites based on geographical regions, ranking status, and program types

**Fig. 2:**
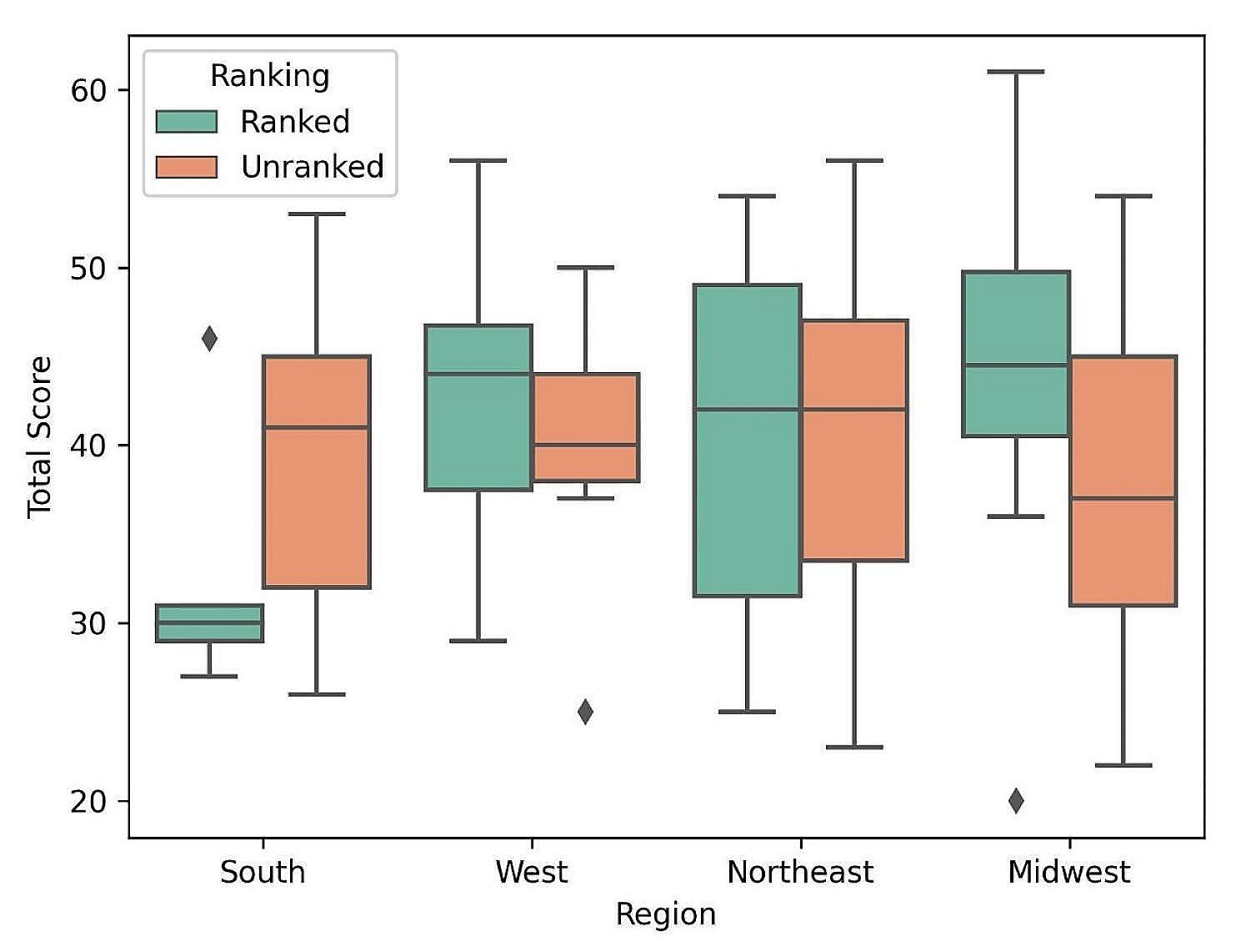
Comparison of total scores for epilepsy fellowship programs across geographic regions. The boxplot illustrates the total scores for epilepsy fellowship programs across four geographic regions, distinguishing programs that are ranked among the top 50 US neurology and neurosurgery programs and unranked programs by color coding. The interquartile range (IQR), representing the range from the first quartile (25th percentile) to the third quartile (75th percentile), is depicted by the box, with the median value (50th percentile) denoted by the center line. Whiskers extend from the minimum to the maximum values, excluding any outliers, which are identified by rhombi. Outliers are defined as points falling more than 1.5 times the IQR above the third quartile or below the first quartile

Table 3 presents the mean and median scores for each category. The scores were assessed for normal distribution using Shapiro-Wilk tests, and it was found that all categories, except education, did not follow a normal distribution. Our analysis indicated no significant variations in scores based on location, academic affiliation, or rank status. Figure 1 demonstrates the average number of criteria mentioned on programs’ websites based on geographical regions, program types, and ranking. A box plot was constructed to analyze the distribution of total scores, as shown in Fig. 2.

**Table 3:** Program details and scores on available information

Category	Max	Mean (std)	Median (IQR)
Recruitment	21	13.06 (2.40)	13 (11–15)
Educational	17	8.16 (2.88)	8 (7–10)
Epilepsy center	9	4.30 (1.95)	5 (3–6)
Compensation	9	4.81 (4.10)	6 (0–9)
Social media	5	3.78 (1.50)	4 (3–5)
Updated information	8	3.39 (1.42)	4 (2–4)

## Discussion

Results of this analysis affirms a deficiency in the information available to fellowship applicants, despite the programs’ online presence. This deficiency significantly impacts the candidates’ experience of applying for fellowship. Having comprehensive and accurate information for fellowship applicants is of utmost importance to ensure a smooth and efficient process and optimal matching between applicants and programs. This system is mutually beneficial: applicants would reduce the stress associated with major life decisions, while programs would attract the most eligible candidates and showcase their strengths, thus preventing any unfilled positions. Anecdotally, applicants use FRIEDA and specific program websites side by side to gain information on their programs of interest. With this in mind, we employed a novel approach by analyzing FRIEDA and specific program websites, providing a more accurate portrayal of the information available to applicants online.

The findings of our analysis of epilepsy fellowship programs information availability indicate that the most commonly encountered features were the program director’s name and email, program description, and program director’s contact number. These findings are consistent with the results of other studies (Khan et al. [7], Hsu et al. [9]). This data allows each program to be identified and establishes a communication channel, bolstering accessibility. Only 41.3% of programs included interview dates, and none of the fellowship websites provided interview day itineraries. Although this finding is not unusual, as Vilanilam et al. reported that itineraries were present in 1.3% of interventional neuroradiology fellowship program websites in 2021 [12], interview day itineraries are important for the recruitment process and significantly influence candidates’ decisions due to travel and scheduling considerations.

Board exam pass rates were typically absent (1.3%) from epilepsy fellowship programs, similar to Khan et al.‘s finding of 4.5% in their examination of cardiology fellowship websites [7]. In contrast, Chu et al.‘s study on anesthesia residency programs revealed that 20% of those programs disclosed their board pass-rates [6]. This may be due to a difference between residencies and fellowships that could be attributed to varying importance placed on board pass rates in distinct stages of training. Only 12.5% of epilepsy programs provided information on legal policies (such as tail coverage). This finding is in sharp contrast to Khan et al.‘s study on cardiology fellowship programs, which found a far higher percentage of 81% for such policies [7]. Application requirements were generally present, but specific information catering to international medical graduates (IMGs) was less frequent. Approximately half of the programs provided visa information. Although it is unclear why some programs exclude this information, being transparent about visa sponsorship policies allows programs to attract compatible applicants. Moreover, organizing information pertinent to IMGs within a dedicated page or subsection would demonstrate a program’s friendliness towards IMGs.

A substantial proportion of programs reported their curriculum (78.8%) and didactics (70.0%). Fellow research or quality improvement (QI) activities were reported by the majority of programs (91.3%). This finding corroborates the emphasis on scholarly activities placed by the residency applicants in a study conducted by Gaeta et al., in which they found that applicants ranked curriculum, information related to the hospital and its affiliates, faculty and resident information, and research as most important to their application [5]. By showcasing research and QI opportunities, programs aim to attract candidates strongly inclined towards evidence-based practice. While 85.0% of program websites listed current faculty members, only 41.3% provided a list of current fellows. This pattern resembles Vilanilam’s study on interventional neuroradiology fellowship program websites, which found faculty listings at 39.2% and current fellow listings at a mere 8.9% [12].

Applicants may seek indicators regarding expected work-life balance, the two most important of which are call schedule and details about fellow life. Both were lacking in our study (29% and 35.5%, respectively). Other features, such as insurance, housing, and meal allowance, could be excellent selling points for programs looking to attract applicants with good compensation and benefits. In their analysis of 84 neuroradiology program websites, Hsu et al. noted that incentives for fellows’ well-being are poorly featured [9].

Considering the detrimental effects of COVID-19 on clinical practice and training in Epilepsy [13], we evaluated the features of epilepsy centers associated with each program. Most programs covered epilepsy monitoring units (EMUs) and epilepsy surgery. Surgical treatment, while not suitable for all epilepsy patients, holds immense promise in transforming patients’ lives. Even though surgical interventions have been proven safe and effective for drug-resistant epilepsy (DRE), only a fraction of eligible patients actually receive them [14]. This underscores the urgency for specialists who can recognize suitable candidates for surgery and oversee their care. Still, other treatments, such as responsive neurostimulation (RNS), deep brain stimulation (DNS), and intra-operative monitoring (IOM), were less frequently mentioned. Only 7.5% of programs mentioned having a preparatory boot camp. The absence of these components may indicate limitations in the training program itself.

The utilization of social media platforms by epilepsy fellowship programs was found to be widespread, with X being the most commonly used platform (88.8%), followed by Facebook (81.3%) and Instagram (71.3%). Furthermore, 78.8% of programs included a functioning link to their program website on at least one of their social media profiles. This highlights social media integration as a tool to drive traffic and provide additional information to prospective applicants. Compared to Pollock et al.‘s study on emergency medicine residency program websites, our findings demonstrate a higher utilization of social media platforms among epilepsy fellowship programs. While X, Facebook, and Instagram were also the most common platforms in their study, the respective usage rates were significantly lower, with X at 15%, Facebook at 12%, and Instagram at 8% [15].

This study has several limitations. First, due to the binary nature of our data analysis, we could not evaluate the ease of website navigation or the quality of information. Second, we examined the content found on program websites and FREIDA webpages without validating how frequently applicants utilize these resources for online program research or whether data from the two sources match up. However, this highlights the need for programs to monitor their published data, ensuring its accuracy continually. Third, conducting a needs assessment of epilepsy fellowship applicants before evaluating websites would have improved this study. Finally, evaluator bias may have influenced the results.

When applicants turn to the internet for information, programs are incentivized to ensure the information they find is thorough, accurate, and up-to-date. This could enhance the matching process and attract highly suitable fellows for each program. To this end, Epilepsy fellowship programs should allocate more resources to manage their program websites.

## Conclusions

In conclusion, our analysis reveals a deficiency in the comprehensiveness of the information available to epilepsy fellowship applicants, impacting their application experience. We identified areas of improvement, based on key shortcomings including interview itineraries, board pass rates, and information catering to international medical graduates. This study emphasizes the value of informative program representations for the benefit of both applicants and institutions, and it further advances the broader discussion on transparency in medical education.

## Data Availability

The datasets used and/or analyzed during the current study are available from the corresponding author on reasonable request.

## Abbreviations

NRMP: National Resident Matching Program
AMA: American Medical Association
ACGME: Accreditation Council for Graduate Medical Education
NAEC: National Association of Epilepsy Centers
EMU: Epilepsy Monitoring Unit
DBS: Deep Brain Stimulation
RNS: Responsive Neurostimulation
IOM: Intraoperative Monitoring
EEG: Electroencephalogram
IMG: International Medical Graduate
QI: Quality Improvement
